# The macroecology of Mesozoic dinosaurs

**DOI:** 10.1098/rsbl.2024.0392

**Published:** 2024-11-13

**Authors:** Alfio Alessandro Chiarenza

**Affiliations:** ^1^ Department of Earth Sciences, University College London, Gower Pl, London WC1E 6BS, UK

**Keywords:** dinosaurs, macroecology, macroevolution, Mesozoic, palaeoecology

## Abstract

Dinosaurs thrived for over 160 million years in Mesozoic ecosystems, displaying diverse ecological and evolutionary adaptations. Their ecology was shaped by large-scale climatic and biogeographic changes, calling for a ‘deep-time’ macroecological investigation. These factors include temperature fluctuations and the break up of Pangaea, influencing species richness, ecological diversity and biogeographic history. Recent improvements in the dinosaur fossil record have enabled large-scale studies of their responses to tectonic, geographic and climatic shifts. Trends in species diversity, body size and reproductive traits can now be analysed using quantitative approaches like phylogenetic comparative methods, machine learning and Bayesian inference. These patterns sometimes align with, but also deviate from, first-order macroecological rules (e.g. species–area relationship, latitudinal biodiversity gradient, Bergmann’s rule). Accurate reconstructions of palaeobiodiversity and niche partitioning require ongoing taxonomic revisions and detailed anatomical descriptions. Interdisciplinary research combining sedimentology, geochemistry and palaeoclimatology helps uncover the environmental conditions driving dinosaur adaptations. Fieldwork in under-sampled regions, particularly at latitudinal extremes, is crucial for understanding the spatial heterogeneity of dinosaur ecosystems across the planet. Open science initiatives and online databases play a key role in advancing this field, enriching our understanding of deep-time ecological processes, and offering new insights into dinosaur macroecology and its broader implications.

## 1. Introduction

**Figure 1.**
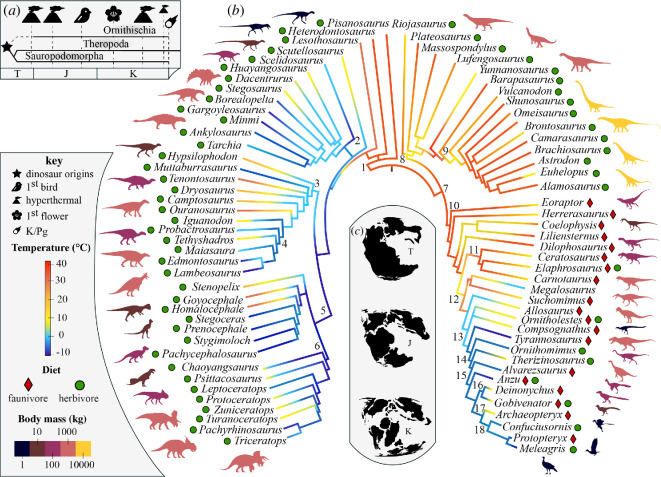
Evolution of macroecological traits in Dinosauria. Large scale event in dinosaur evolution (*a*); the origin of dinosaurs (star), hyperthermals (volcano), the earliest fossil Avialae (bird), the earliest fossil angiosperm (flower), the Cretaceous/Palaeogene mass extinction (asteroid). Phylogeny of dinosaurs (*b*) redrawn from Sereno [2] and adapted to the current consensus and upon which an ancestral state reconstruction of temperature niche (mean annual temperature) after Chiarenza *et al*. [4] is plotted; Mesozoic palaeogeographies (*c*) for Triassic (T), Jurassic (J) and Cretaceous (K). Silhouette colours symbolize body mass for each of the taxa represented (after [5]); information on dietary habits are plotted after Barrett [6] and Zanno & Makovicky [7]; numbers represent clades discussed through this study: 1, Ornithischia; 2, Thyreophora; 3, Ornithopoda; 4, Hadrosauroidea; 5, Marginocephalia; 6, Ceratopsia; 7, Saurischia; 8, Sauropodomorpha; 9, Sauropoda; 10, Theropoda; 11, Ceratosauria; 12, Tetanurae; 13, Coelurosauria; 14, Maniraptoriformes; 15, Maniraptora; 16, Deinonychosauria; 17, Avialae; 18, Ornithothoraces. Palaeogeographies modified from original plots via R package ‘mapast’ [8] using plate models by Scotese [9].

For over 160 million years (from approximately 230 to 66 million years ago, Ma), Earth’s terrestrial ecosystems were dominated by dinosaurs [1]. These organisms diversified into a remarkable array of ecological niches, evolving diverse dietary preferences [2] and occupying a broad spectrum of body sizes [3] (figure 1), rivalling other terrestrial vertebrate groups, and posing intriguing questions about the ecology of Mesozoic ecosystems. How did multiple whale-sized terrestrial herbivores coexist in the same ecosystem [10], and how could primary productivity remain stable under such intense browsing pressure [6]? Did several species of giant theropod carnivores share high trophic levels simultaneously [11]? Did dinosaur body size evolve, or their diversity increase, in response to the unique climatic regimes of Mesozoic Earth systems [12]? These and many other ecological questions seem insurmountable due to the vagaries of an incomplete fossil record, which nonetheless reveals a high disparity in diversity and ecologies that evolved over a 160 million-year interval.

**Figure 2.**
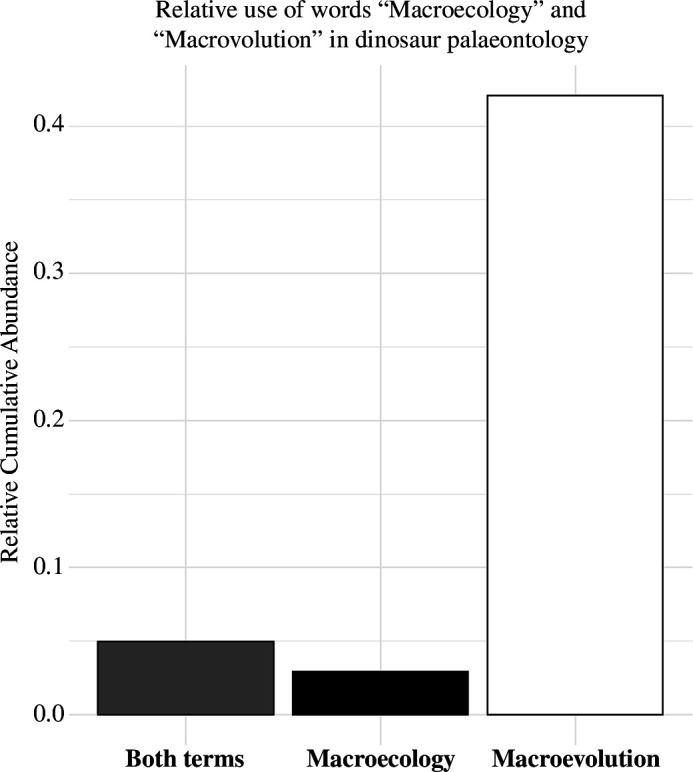
The use of macroecology in dinosaur palaeontology. Relative cumulative abundance of the use of the terms ‘macroecology’ and ‘macroevolution’ in dinosaur palaeontological literature since 1989. Data downloaded from Google Scholar (https://scholar.google.com/) on 05/04/2024.

While twentieth century palaeontological research focused on large-scale evolutionary processes (macroevolution), increased global data coverage with high sampling in some exceptional areas [13–15] now allows the investigation of the drivers of spatial patterns [16], a ‘deep-time’ macroecological enterprise. Although its origins date back to the nineteenth century [17–19], macroecology is a 35-year-old subdiscipline of ecology focusing on large-scale processes like climate and geography shaping biodiversity patterns. Macroecology, a term coined in 1971 [20], has seen limited application in palaeontology since its scientific formalization [21], often being paired with macroevolution (figure 2). Macroecology and macroevolution are often conflated in the scientific literature due to their interconnectedness, although macroevolution addresses prevalently temporal patterns (e.g. timing of clade diversification), while macroecology emphasizes spatial patterns (responses of biodiversity to broad Earth system interactions and feedback) [22].

While macroecology is prevalent in Quaternary palaeoecology [23], it is rarely used alone by dinosaur palaeontologists, despite its first use in this subject dating back to right after its formalization [24]. As a major component of their environments in terms of diversity and biomass [2,25], dinosaurs influenced various ecological processes, affecting other non-dinosaurian species. Their global distribution, extensive temporal range and role in understanding modern birds make them crucial for studying macroecology in deep time, broadening the scope of a science limited by present-day perspectives and an anthropogenically depauperated Quaternary record.

In the last four decades, there has been an exponential increase in dinosaur studies, with new species described at an average rate of two per week [26]. This constant update continuously challenges our understanding of dinosaur palaeobiology and Mesozoic ecosystems. The growing dataset of new taxa, localities and phenotypic diversity has allowed palaeontology to transition from an idiographic (descriptive and data collection) phase to a nomothetic one, in which principles relevant to evolutionary theory, including spatial patterns and their bearing in understanding the effects of large scale (e.g. climate and geography) on biodiversity, are formulated and tested against the empirical record [27].

Here, I outline the main topics investigated in recent years in non-avian dinosaur macroecology, including their evolutionary origins, ecosystem structure, the geographic factors influencing their diversity and trait distribution (e.g. trends in body size, diversity and reproductive traits across different regions and climates; table 1), and recent methodological advances enabling such studies. I then discuss and propose new perspectives for developing a methodologically robust and epistemologically grounded research programme on dinosaur macroecology moving forward.

**Table 1.** Summary of main macroecological principles investigated in dinosaur palaeontology with key taxonomic, regional and temporal examples from the literature.

macroecological principles	description	case studies
Allen’s rule	in homeothermic animals living in cold environments, a reduced ratio of body surface area to volume helps them retain heat more efficiently (shorter and stouter appendices).	Late Campanian–Early Maastrichtian Hadrosauridae in North America [28].
Bergmann’s rule	homeothermic animals in colder climates are typically larger than those in warmer regions, which helps them conserve heat more effectively.	Late Campanian–Early Maastrichtian Troodontidae [29]; Ornithischia and Theropoda in the Campanian–Maastrichtian of North America [12].
Damuth’s law	inverse relationship between body size and population density: as the size of a species increases, the number of individuals living in a given area decreases.	Maastrichtian of North America, *Tyrannosaurus rex* [30].
ecological release	population growth and increased diversification result from the relaxation of environmental limiting factors, such as competition, which allows for expansion into new niches and ecomorphological space.	Late Triassic Dinosauria versus non-dinosaur tetrapods (global) [31–34].
island rule (Foster’s rule)	animals isolated on islands often experience changes in body size, with smaller species tending to become larger (gigantism) and larger species tending to become smaller (dwarfism).	*Europasaurus,* Kimmeridgian (Europe) [35]; titanosaurs in Europe [36,37] and South America [38]; *Tethyshadros insularis*, Campanian of Europe [39,40].
latitudinal biodiversity gradient (LBG)	species richness peaks in the tropics and declines progressively toward the poles.	Jurassic–Cretaceous Dinosauria (global) [41,42].
thermogeochromic egg variation (TEV)	in birds from cold habitats, especially those with nests exposed to direct sunlight (and coinciding with high latitudes), eggshells are darker to enhance heat absorption; eggshells tend to be lighter (e.g. light blue and green) in the tropics.	modern examples [43], with future potential for investigation in the dinosaur fossil record [44,45].
species–area relationship (SAR)	species richness increases in proportion to the available area, with larger areas supporting greater biodiversity.	Maastrichtian Dinosauria (global) [46].

## 2. Origins and ecosystem structure of dinosaur ecosystems

### (a) Competition, ecological release and the rise of dinosaurs

Dinosaurs originated in the Late Triassic, with early Sauropodomorpha and Theropoda appearing around 230 Ma in South America [47]. Earliest ornithischian occurrences come from the Hettangian–Sinemurian (201−193 Ma) of Gondwana [48,49]. Some phylogenetic analyses suggest that Silesauridae might represent early ornithischians, possibly extending the presence of this main dinosaurian subclade and in turn pushing back dinosaur origins to the Middle Triassic [50]. Initially, dinosaurs were small [5], bipedal and less ecologically significant compared with other archosaurs and synapsids [31]. Their diet varied from carnivory to herbivory [51]. It remains unclear how dinosaurs succeeded in becoming the dominant archosaur lineages after the end-Triassic extinction. In terms of species richness (mere count of unique taxa), dinosaurs were less diverse than crurotarsans (crocodile-line archosaurs) in Late Triassic ecosystems [32]. If ecological niches are approximated with ecomorphological diversity, dinosaurs occupied a noticeably reduced ecospace compared with crurotarsans [31]. This ecological imbalance was reversed in the Early Jurassic (from Hettangian to Toarcian), with the ecomorphological diversity of dinosaurs surpassing that of the crurotarsans. A gradual ‘competitive exclusion’ model, with dinosaurs slowly outcompeting other archosaur clades through the Triassic–Jurassic transition, seems unlikely [33,34]. Instead, opportunism in the wake of the end-Triassic extinction as a process for their ecological release at the dawn of the Jurassic is currently favoured [33]. Extrinsic factors such as climate might have acted as a main constraint on dinosaur ecological release. Olsen *et al*. [52] discussed evidence for volcanically generated cold-pulses interspersed with the longer term volcanic hypsithermal event at the Triassic/Jurassic boundary as a possible extrinsic filter selecting the climatically more adaptable dinosaurs at the expense of more stenothermal non-dinosaurian tetrapods.

Evaluating ecologically relevant traits through functional morphology can reveal how some traits may have conferred advantages during this key transition, highlighting the selective roles operated by various environmental or biotic factors (see García-Girón *et al*. [53] for an end-Cretaceous analogue). Integrating functional ecology with machine learning, as performed in Foster *et al*. [54] to test extinction selectivity in invertebrates, can provide new methods to explore hypotheses explaining the rise of dinosaurs.

### (b) Dinosaurs and primary productivity

The lower trophic levels of dinosaur ecosystems were composed of plant groups partially not comparable with modern ones. Mesozoic primary productivity included cycadophytes, gymnosperms, ferns, horsetails and ginkgoes, likely representing the main food source for herbivorous dinosaurs [6,55]. Herbivorous dinosaur dietary preferences have been explored via insights from animal nutrition science and comparative physiology on modern herbivores’ digestive tract. As bulk feeders [56], sauropods likely relied on plants providing substantial biomass and rapidly regenerating foliage, like conifers and ginkgoes, over less nutritious ferns and cycads. The distribution of plant biomes influenced herbivorous dinosaur distribution and diversity across different latitudes. Mesozoic conifers, evolving into frost-tolerant specialists, thrived in high-latitude regions, as evidenced by fossil records from polar regions [57]. Their abundance at high latitude suggest they could support the dietary needs of giant dinosaurian foragers at all latitudes, including polar biomes [4]. Angiosperms provided a succulent and poorly chemically defended food source [6,55], and their mid-Cretaceous rise coincides with their radiation and the decline of gymnosperms [58], particularly at lower latitudes [57]. A direct macroevolutionary response in dinosaurs to the appearance of angiosperms has been hypothesized [59], but quantitative assessments have found limited support for this interpretation [60–62]. The rise of angiosperms has been suggested as a cause of sauropod decline, at least in the Northern Hemisphere [60] while the more complex dental batteries of later diverging neornithischians (e.g. hadrosaurs and ceratopsians) might have provided an advantage for angiosperm consumption [59,61]. Detailed microwear [63,64] or isotopic studies [16,65] may shed new light on these clades’ preferential habits in plant consumption.

### (c) Co-occurrence and spatial patterns

Collective aggregation or solitary habits in animals affect various aspects of terrestrial ecology, from the extent of occupied areas to migratory behaviour in response to resource consumption and depletion [25,66]. Evidence from ichnology and bonebeds suggests that many dinosaur groups, such as ceratopsians [67,68], ornithopods [69,70], theropods [71,72] and sauropods [73–75], may have been gregarious. This social behaviour likely served as an adaptation for protection against predators, efficient foraging and enhanced breeding success [76]. Parallel trackways with consistent depth, direction and lacking superposition suggest herding behaviour [77], though some may represent solitary dinosaurs moving in the same direction at different times due to geographical constraints [78,79]. Theropod trackways may indicate gregarious behaviour [80–82] in predatory dinosaurs, but the social nature of theropods remains debated [83,84]. Several monospecific bonebeds, previously considered evidence of gregarious aggregations, are more likely predator traps [85]. While body fossils provide useful taxonomic and population composition details, skeletal evidence must be carefully examined within its taphonomic context to accurately interpret dinosaur social behaviour [86].

Spatial distribution of dinosaur ichnofacies from the upper Pliensbachian of Poland, suggest closer association for early ornithischians to the coastlines, co-occurring with small theropods, while larger theropods and sauropomorphs are more abundantly found inland [87]. Brinkman *et al*. [88] observed an increase in ceratopsid occurrences and a decrease in ankylosaurs and pachycephalosaurs towards the palaeoshoreline, attributed to clade-specific environmental segregation [88,89]. Abelisaurids and carcharodontosaurids in South America share distributions with titanosaur sauropods. In the Western Interior, tyrannosaurid distributions closely parallel those of herbivores, with albertosaurine tyrannosaurids co-occurring with centrosaurine ceratopsids and lambeosaurine hadrosaurids, while tyrannosaurines co-occurred with chasmosaurine ceratopsids and hadrosaurine hadrosaurids [90].

Butler & Barrett [91] used correlative statistics to investigate the environmental preferences of Cretaceous herbivorous dinosaurs based on their spatial distribution. They found that nodosaurid ankylosaurs and hadrosaurids are strongly associated with marine sediments, while ceratopsians, pachycephalosaurs, theropods, sauropods and ankylosaurine are more common in terrestrial sediments. This supports the hypothesis that nodosaurids (although see Arbour *et al*. [92] for some caveats) and hadrosaurids were more prevalent in coastal or fluvial environments, while marginocephalians and ankylosaurids preferred inland habitats [93]. Quantitative studies of predatory theropod habitat preferences have shown that spinosaurids were predominantly associated with coastal palaeoenvironments, supporting a primarily piscivorous diet [94]. By contrast, Abelisauridae and Carcharodontosauridae are associated with terrestrial habitats, with abelisaurids being more common in inland areas compared with spinosaurids and carcharodontosaurids. Lyson & Longrich [95] used lithologies as proxies for palaeoenvironments to test habitat preferences in North American Maastrichtian dinosaurs. Their findings suggested ceratopsians preferred habitats away from rivers in coastal lowlands, while ornithopods favoured habitats closer to riverine systems. *Tyrannosaurus rex*, the primary secondary consumer in these terminal Cretaceous assemblages, is associated with both environments, indicating a generalist diet and extensive coexistence with various herbivores.

## 3. Large-scale patterns in dinosaur macroecology

### (a) Geographic influence on dinosaur diversification

The Jurassic radiation and diversification of major dinosaur lineages may have been driven by continental fragmentation resulting from the breakup of Pangaea, which affected the available area for species [96,97] (figure 1*c*), vicariance and access to landmasses [98]. The species–area relationship (SAR) indicates that species richness scales with increasing available area, an ecological principle relevant to various terrestrial and marine systems [99]. Habitat fragmentation can either limit available area, negatively affecting diversity, or enhance isolation and speciation, thereby promoting diversity. These principles have been integrated into the species-fragmented area relationship (SfAR) [100]. The fragmentation of Pangaea since the Late Triassic, with the Atlantic Ocean spreading and sea level transgression inundating continental crust [101], particularly in the Late Cretaceous (figure 1*c*), led to isolation and continental endemicity, as seen between Northern Laurasian and Southern Gondwanan faunas [102]. This contrasts with the more uniform and cosmopolitan communities of the Late Triassic to Middle Jurassic [102–104]. Due to its generalization and wide applicability, SAR has been used in dinosaur palaeontology to estimate species numbers during specific intervals and understand the impact of changing terrestrial area extent on diversification. Dinosaur species richness has been modelled to largely track SAR [105] throughout the Mesozoic. However, principles of island biogeography [98] also predict increased species diversity due to enhanced isolation. Results from SAR modelling are susceptible to undersampling, leading to an underestimation of standing diversity in several large areas [46,106]. This underestimation has significant implications for understanding critical changes in diversity due to large-scale processes such as long-term climatic fluctuations and extinction events [106].

### (b) Macroecological rules in dinosaur palaeobiology

The impact of environmental agents on biodiversity at the macroscale has been quantified into first-order macroecological rules (table 1), describing features like the variation in species richness and body size within abiotic and biotic domains. At the intersection of area extent and climate are discussions about the latitudinal biodiversity gradient (LBG) and its establishment in deep time [107]. The LBG, characterized by a peak in species richness in the tropics that declines towards the poles, stands as a fundamental pattern in modern biodiversity [108,109]. Explanations for this phenomenon include biological and abiotic drivers, such as gradients in temperature, light, seasonality and differential speciation rates associated with latitude, suggesting climate as the primary driver [108,110–112]. Conversely, some arguments propose the larger land availability at the equator compared with the poles, implying a SAR effect, to explain the LBG [108,113,114].

Deep-time investigations on LBG encompass marine invertebrates [115,116], plants [117], insects, turtles, crocodylomorphs, Cenozoic mammals and non-avian dinosaurs [41,118–121]. Contrary to the modern LBG, Mesozoic dinosaur studies suggest a diversity peak at temperate latitudes, with a ‘relaxed’ LBG, particularly during the Late Cretaceous [41]. Geographic partitioning was recovered between major herbivorous dinosaur taxa, with sauropod diversity peaking at lower palaeolatitudes and ornithischian diversity at higher palaeolatitudes. The combined influence of equable climates and land area likely dictated species distribution in the Mesozoic. Climatic factors influenced sauropod distribution similarly to crocodylomorphs [122,123], with preferences for tropical and subtropical zones [124–126]. These patterns may reflect thermophysiological differences between dinosaur subclades [4]. Despite Mesozoic dinosaur records contradicting a modern-type LBG, fossil bias must be considered. Global fossil data are crucial for understanding LBG’s temporal dynamics, but Mesozoic spatial patterns pose challenges due to the predominantly mid-latitudinal spread of fossil localities [127]. Close *et al*. [128] highlighted the limited palaeolatitudinal band available for vertebrate fossil sampling (between 30° and 60°). Fossil-bearing localities beyond this range typically yield singleton taxa but show equal sampling coverage independently of the systematic group, particularly at high latitudes [42]. Heterogeneous sample compositions across latitudinal bands [129] may obscure recognizing a modern-style LBG, impeding the estimation of correlations between species richness and environmental proxies. Nevertheless, rare fossiliferous localities from latitudinal extremes and thorough taxonomic sampling provide valuable references for reconstructing past environmental conditions [90,130–133] and biogeographic trends [134]. Understanding these climatic constraints and accounting for sampling artefacts are crucial for elucidating the abiotic drivers that shape latitudinal distribution and affect LBG configuration [110].

Spatial variation in dinosaur body-size is another deep-time macroecological pattern worth investigating. Bergmann’s rule posits that homeotherm animals in colder climates tend to be larger than their counterparts in warmer regions. To verify this rule, Fiorillo [29] took an empirical approach, focusing on troodontid dinosaurs in North America, particularly analysing tooth size as a proxy for body size, finding northern troodontids to be bigger than southern relatives. This method offers valuable insights due to the relatively higher abundance and preservability of dental material compared with other skeletal elements. It is crucial though to consider the taxonomic scale when applying Bergmann’s rule. While this pattern may hold true for closely related populations with similar phenotypic and ecological characteristics, its validity diminishes when applied across ‘higher ranked’ clades (e.g. supraspecific systematic groups). Wilson *et al*. [12] advocated for phylogenetically informed statistical models to test Bergmann’s rule, finding limited support for a correlation between dinosaur body size and temperature. However, these models, operating above the species level, may overlook intraspecific variation at which the rule is defined. A nuanced interpretation suggests that Bergmann’s rule might better suit lineage-level analyses rather than species-level assessments. Approaches like those by Wilson *et al*. [12] follow this rationale, highlighting general trends and describing spatially explicit relationships due to the increasing availability of fossil occurrences and palaeoclimate models for the Mesozoic. This approach has the virtue of overcoming the constraints of bias obscuring sub-generic diversity and mitigating it with a phylogenetic inference-guided approach. Studies by Blackburn *et al*. [135] and Meiri [136] support this perspective, focusing on closely related taxa inhabiting cooler climates, and highlight the future promise of following this example.

Complementary to Bergmann’s rule is Allen’s rule, which states that the body-surface area to volume ratio is minimized in homeothermic animals living in cold environments. Although no quantitative verification of this rule in the Mesozoic dinosaur record has been performed, some relevant observations can be reported. An implication of Allen’s rule is that the surface area of appendages (such as tails, crests or limbs) is reduced to minimize heat dissipation in homeotherms adapted to cold environments. Assuming at least two lineages of dinosaurs as homeotherms (Ornithischia and Theropoda [4]), they would be expected to have shorter, slimmer tails and limbs, conferring a somewhat stockier appearance to their body plan, in cold settings. Fiorillo & Gangloff [28] documented several predominantly juvenile individuals from the Liscomb bonebed in the Prince Creek Formation of Alaska to test the hypothesis that hadrosaurs from these latitudes were seasonal migrants. A corollary of their investigation, which excluded such a hypothesis, was the assessment of similar body proportions (both in juveniles and adults) compared with those of more southern relatives (e.g. *Maiasaura* [137] and *Edmontosaurus* [138]). This implied a lack of variation in limb-to-body proportions on a latitudinal gradient, representing an—admittedly loose—but probably the best available to date, test of Allen’s rule in Mesozoic dinosaurs. More complete remains of polar tyrannosauroids [139], hadrosaurs [140,141] and ceratopsians [142] are welcome to further test this hypothesis.

The island rule [143] is a macroecological principle describing how isolated animals on islands often undergo significant changes in body size, with smaller species tending toward gigantism and larger species toward dwarfism (table 1). Initially identified in modern mammals and documented in Quaternary ones, such as Mediterranean dwarf elephants, this pattern has been observed across vertebrate groups, including Mesozoic dinosaurs [32]. Insular dwarfism in non-avian dinosaurs, however, requires careful evidence beyond mere size comparisons with larger relatives, including histological markers of reduced growth rates and osteological signs of maturity (e.g. an external fundamental system [144]) alongside a robust tectonostratigraphic framework confirming insular environments. For instance, insular dwarfism has been well-documented in the sauropod *Europasaurus* from the Kimmeridgian Lower Saxony Basin of Germany [35]. By contrast, the hadrosauroid *Tethyshadros insularis* from the Campanian Liburnian Formation of Italy, once thought to be an insular dwarf for its smaller size compared with later diverging hadrosaurids [39], lacks osteohistological maturity signals. Recent revisions of its stratigraphic context [40] challenge the original interpretation, arguing for a less insular palaeoenvironment. These revisions suggest that the small size of *Tethyshadros* fits within the broader size range of early diverging hadrosauroids, compared with their later, larger relatives (e.g. North American and Asian latest Cretaceous hadrosaurids [145]).

Variation in egg colours and other shell features with latitude (thermogeochromic egg variation, TEV from now on; table 1) has been documented for many clades of birds and has been investigated under a macroevolutionary lens by Wisocki *et al*. [43]. We now know that the pigmentation in bird eggshells, once believed to be a unique avian trait, actually originated from non-avian dinosaurs [44]. The implications of studying egg colour and shell morphology in the dinosaur fossil record [45] may open new scenarios in investigating the relationships between the environment and these reproductive traits. The morphology and geographic distribution of fossil eggs may offer clues about nesting habits and environmental adaptations in Mesozoic dinosaurs [146]. Pigment analyses of maniraptoran eggs highlights that coloured eggs may have evolved alongside open nesting behaviours and brooding [146], likely serving as camouflage against predators and enabling nesting site recognition, as seen in modern birds. Other clades, like sauropods, built sandy in-filled hole nests (like modern marine turtles) that relied on solar or geothermal heat for incubation [146,147]. Furthermore, the identification of green, blue and speckled eggs may suggest both ecological and thermoregulatory functions in dinosaurs inhabiting different climates, highlights adaptations to different environments as seen in birds today, and a potential adherence to TEV in non-avian dinosaurs.

Damuth’s law relates large body sizes to low population density in land mammals. Marshall *et al*. [30] applied this principle, using a power law with proxies from palaeobiogeography and palaeobiology to estimate the overall population density of the theropod *Tyrannosaurus rex* during its approximately 2.5 million-year stratigraphic range. Despite uncertainties in parameters like areal extent and metabolism, they provided a rough estimate: up to 2.5 billion *T*. *rex* individuals may have ever lived, with a fossil recovery rate of about 1 per 80 million individuals, or 1 per 16 000 in areas of higher sampling. The remarkable aspect revealed in this study is not the absolute numbers provided, but the uncertainty bracket and extremely low sample size for one of the better-known dinosaurs. This highlights the severe limitations palaeontologists face to investigate large-scale macroecological patterns from even more incomplete palaeobiological samples.

## 4. Future directions

To shape the future of dinosaur macroecology, we must first address the limitations imposed by an incomplete and patchy vertebrate fossil record, especially in terrestrial environments. This necessitates scrupulous sampling strategies and careful extrapolation of ecological trends and patterns over time and space. To improve our analytical approaches and investigation of patterns in dinosaur biology and palaeoecology throughout the Mesozoic, several key recommendations emerge.

First, improving chronostratigraphic frameworks will help distinguish between contemporaneous and time-averaged assemblages, clarifying species interactions and ecological dynamics. This could involve more refined use of advanced radiometric dating techniques (e.g. tracing zircon-rich crystals in volcanoclastic layers through sedimentary successions [148] or from apatite in fossil bones using laser-ablation coupled with plasma-mass spectrometry [149] for U–Pb dating [148,150]); use of Bayesian age-stratigraphic modelling [151] (for constraining the age of the site from multiple data points) and extensive biostratigraphic and lithostratigraphic correlations. The presence of geochronologists [150,152] in any field enterprise is recommended, as it would enable palaeontologists to construct more accurate timelines of dinosaur communities. Time-averaging is a significant issue affecting palaeoecological chronologies, in which the mixing of skeletal elements from different chronological horizons can distort perceptions of palaeocommunity synecology [153]. The ‘mid-Cretaceous’ Kem Kem Compound Assemblage, with its presumably overcrowded guild of predators—known as ‘Stromer’s riddle’—demonstrates how separating different facies into distinct chronological horizons can clarify similar palaeoecological paradoxes [11,154]. Improved chronostratigraphic frameworks can also elucidate our understanding of multiple sauropod species coexistence in the Morrison Formation [10,15]. Enhancing stratigraphic and spatial contexts through the integration of sequence stratigraphy, sedimentology and taphonomical observations is crucial [10,15,152,155,156].

Second, thorough taxonomy and appropriately based systematics, solidly grounded in up-to-date and carefully considered anatomical characters, are the foundation of any community-level reconstruction and palaeoecological consideration. For instance, taxonomic re-evaluations, like the synonymy of ‘*Nanotyrannus*’ with *Tyrannosaurus rex*, affect our understanding of predator–prey dynamics and niche partitioning [157,158] in latest Cretaceous North American ecosystems [159]. Overzealous conservatism in species lumping and uncertainty in species delimitation have likely forced artificially low levels of alpha-diversity in many dinosaur-dominated assemblages. Conversely, ascribing variation to taxonomic distinction that might be ontogenetic would overinflate alpha diversity. Future refinements in species-level identification in dinosaur palaeontology will likely overhaul palaeodiversity in these study systems, restructuring our reconstructions of their palaeoecology.

Third, adopting advanced statistical methods and computational tools to analyse fossil data can quantitatively support inferences on the direct or indirect effects of the abiotic environment on dinosaur ecology [61,62]. Statistical investigations emphasize the significant role of sampling in diversity reconstructions, though further studies are needed to address the influences of sedimentation and fossil accumulation rates [128,160]. Computational methods, such as those developed by Close *et al*. [128], attempt to mitigate these biases using minimum-spanning trees and subsampling techniques. Approaches that emphasize the uncertainty bracket over absolute metrics are highly valuable, highlighting time intervals [161] or areas [42,106] where the record should be more carefully scrutinized for distortive agents rather than relying on absolute but labile measured signals. Techniques such as machine learning [162–164], network analysis [165] and Bayesian inference [166–169] offer promising avenues for corroborating hypothesis testing in palaeodiversity and macroecology, providing a more robust quantitative framework than ever before. This is similar to how quantitative systematics approaches in the 1970s provided replicable, numerical tools to evolutionary biologists, improving the methods used to reconstruct dinosaur evolutionary trees. Besides reconstructing ancient ecosystems with greater accuracy, these methods can handle large datasets and complex variables, providing more robust models for testing hypotheses on dinosaur ecology and co-evolution with the Earth System [4,13,42,53,54,106,170].

Fourth, large, open-source fossil occurrence databases, such as the Paleobiology Database (paleobiodb.org), along with advances in computational power and analytical techniques, have renewed interest in addressing biases and conducting macroecological studies in deep time. Invertebrate palaeontology [171–174] has pioneered many of these approaches, which have since been applied to vertebrate palaeontology [175–180] and, more recently, dinosaur palaeontology over the last two decades (e.g. [41,42,181]). This wealth of data is crucial for quantifying broad patterns of dinosaur diversity and palaeobiology through time and space. Ensuring the accuracy of stratigraphic and taxonomic data (e.g. [4,129,161]) within these occurrences is essential, and these databases should be seen as evolving systems that benefit from continuous input and refinement. Just as with museum collections, data from online fossil occurrence databases should be carefully vetted and cross-referenced. Researchers are encouraged to apply their own expertise (chronological, regional, taxonomic), as well as collaborate with colleagues with compatible expertise, to ensure that the data they analyse are reliable and robust. Initiatives like the Paleobiology Database should be expanded and further integrated with new digital tools (macrostrat.org for stratigraphic [182], morphobank.org for morphological and gbif.org for biodiversity data) and research platforms/consortia (e.g. Palaeoverse [183]) can streamline these research efforts. A use of database does not just provide ready to access information to scholars, but also improve wider accessibility and replicability of results, other than allowing a wider community to cross-check, verify and implement such sources of information for the benefit of the broader community. Integrating sequence stratigraphy [155,184,185] into palaeontological models may better advance our understanding of how sedimentation rates and facies deposition influence diversity signals, separating chaotic and random mixing of communities to identify only those taxa truly in sympatry that might have been interacting in their original, pre-diagenetic biocoenoses.

Fifth, extensive and targeted fieldwork in under-sampled continents, regions and palaeoenvironments is crucial to observe the heterogeneity in spatial patterns of palaeodiversity. Fieldwork should be strategically planned to cover diverse habitats and geological settings, ensuring a more representative sample of dinosaur life through different Mesozoic intervals.

Lastly, interdisciplinary approaches that integrate sedimentology, geochemistry and palaeoclimatology can offer deeper insights into the environmental contexts in which dinosaurs lived. Understanding the interplay between climate, vegetation and dinosaur communities, with a synergic integration of efforts from vertebrate palaeontologists, palaeobotanists and physical geoscientists (e.g. sedimentologists and stratigraphers) will shed light on their adaptive strategies and responses to environmental changes (e.g. feeding preferences and flexibility, shifting growth rates, thermophysiology, size variation [4,42,186,187]). Advanced analytical techniques, such as stable isotope analysis, biomechanical and palaeoclimate modelling, are essential for understanding the complex interactions between abiotic factors and these traits [188]. By examining patterns of body size, dietary requirements, population density and spatial distribution, palaeontologists can elucidate the niche dynamics and trophic structures of dinosaur-dominated communities in the Mesozoic [13,189,190]. Future research should focus on refining these methods and developing phylogenetically informed statistical methods and spatially explicit models to test macroecological hypotheses [4,12].

## 5. Conclusions

Studying macroecological principles applied to the dinosaur fossil record enhances our understanding of evolutionary and ecological processes over geological time. Fostering a more collaborative, open science ecosystem will increase public engagement and education about the importance of palaeontological research, garnering support and funding for future investigations. Public interest in dinosaurs is already high, and leveraging this fascination through outreach programmes and citizen science projects can boost resources for addressing key palaeoecological questions. Dinosaur macroevolution was essentially a 160 million-year natural experiment on the effects of dramatic geographic and climatic changes [34,130,146,191], impacting terrestrial ecosystems at multiple levels, which we can only document through palaeontology. By elucidating the dynamics of ancient ecosystems and the factors driving biodiversity patterns, palaeontologists can provide valuable insights into how organisms respond to environmental changes, equally generating broader interest in the scientific community and among the general public. Leveraging this acquisition, deep-time macroecology can direct public fascination with dinosaurs towards understanding the impact of climate change on ecosystem evolution. This is crucial for our modern society and underscores the role of dinosaur palaeontologists in addressing these issues.

This review aims to guide those interested in a richer understanding of ancient terrestrial ecosystems. By addressing the challenges here presented and developing new technologies and methodologies to tackle them, we can advance dinosaur palaeoecology and its broader impact on our knowledge of natural history and societal relevance.

**Figure 3.**
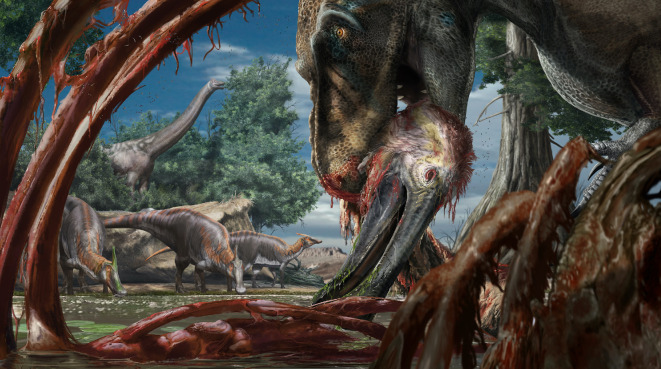
Dinosaur ecology in the Nemegt Basin (Upper Cretaceous of Mongolia). A *Tarbosaurus* is consuming a *Deinocheirus* carcass, imposing a distortive, first-order taphonomic filter for interpreting the palaeoecology of this community (the hadrosaurid *Saurolophus* drinking opposite the tyrannosaurid and the sauropod *Nemegtosaurus* foraging in the background). Reconstruction by Davide Bonadonna.

## Data Availability

This article has no additional data. Supplementary material is available online [192].
